# Reference interval, longitudinal variability and reliability of activated clotting time in healthy dogs using a point‐of‐care analyser

**DOI:** 10.1002/vms3.1148

**Published:** 2023-05-04

**Authors:** Arnaut Hellemans, Nausikaa Devriendt, Filip De Somer, Sofie Marynissen, Sylvie Daminet, Dominique Paepe, Pascale Smets

**Affiliations:** ^1^ Small Animal Department, Faculty of Veterinary Medicine Ghent University Merelbeke Belgium; ^2^ Department of Cardiac Surgery, Faculty of Medical Sciences Ghent University Ghent Belgium; ^3^ Experimental Research Laboratory of Cardiac Surgery and Circulatory Physiology, Faculty of Medical Sciences Ghent University Ghent Belgium

**Keywords:** bedside testing, canine, coagulation, heparin monitoring, i‐STAT 1, screening

## Abstract

**Background:**

Activated clotting times (ACTs) are used to screen for coagulopathies and monitor heparin therapy.

**Objectives:**

To determine a reference interval (RI) for ACT in dogs using a point‐of‐care analyser, to quantify intra‐subject within‐ and between‐day variability, to quantify analyser reliability and inter‐analyser agreement and to study the influence of a delay in measurement.

**Methods:**

Forty‐two healthy dogs were included. Measurements were performed on fresh venous blood using the i‐STAT 1 analyser. The RI was determined using the Robust method. Intra‐subject within‐day variability and between‐day variability were quantified between baseline and 2 h (*n* = 8) or 48 h (*n* = 10) later. Analyser reliability and inter‐analyser agreement were studied by duplicate measurements (*n* = 8) on identical analysers. The influence of measurement delay was studied before and after a delay of one analytical run (*n* = 6).

**Results:**

Mean, lower and upper reference limits for ACT were 92.9 ± 9.1, 74.4 and 111.2 s, respectively. Coefficients of variation of intra‐subject within‐ and between‐day variability were 8.1% and 10.4%, respectively, resulting in a significant between‐day measurement difference. Analyser reliability assessed by the intraclass correlation coefficient and coefficient of variation were 0.87% and 3.3%, respectively. Significantly lower ACT values were observed after a measurement delay compared to direct analysis.

**Conclusions:**

Our study provides an RI for ACT in healthy dogs using the i‐STAT 1 and suggests low intra‐subject within‐ and between‐day variability. Analyser reliability and inter‐analyser agreement were good; however, analysis delay and between‐day differences could significantly influence ACT results.

## Graphical abstract

Activated clotting times (ACTs) are used to screen for coagulopathies and monitor heparin therapy during intravascular procedures. Our study provides an RI for ACT in healthy dogs using the i‐STAT 1 and suggests low intra‐subject within‐ and between‐day variability. Analyser reliability and inter‐analyser agreement were good; however, analysis delay and between‐day differences could significantly influence ACT results.

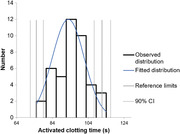

## 1 INTRODUCTION

Activated clotting time (ACT) is a valuable screening test and alternative to activated partial thromboplastin time for the detection of coagulation disorders in dogs and cats involving the so‐called intrinsic and common pathways (Hyatt & Brainard, 2016; See et al., 2009; Tseng et al., 2001). In addition, it allows to monitor anticoagulant therapy with unfractionated heparin in dogs, cats and humans during various intravascular procedures, most commonly those requiring cardiopulmonary bypass or left heart catheterisation (Griffiths 2010; Hyatt & Brainard, 2016; Pelosi et al., 2013; Thompson et al., 2019; Uechi 2012).

ACT assays were initially performed by manually mixing fresh non‐coagulated whole blood in a test tube with a contact activator such as diatomaceous earth or kaolin at 37°C. ACT was defined as the time duration in seconds between contact activation and visual clot formation (Byars et al., 1976; Middleton & Watson, 1978). Contemporary point‐of‐care (POC) analysers automate the detection of fibrin formation using mechanical or photo‐optical sensors, which has improved practicality and measurement accuracy (Thompson et al., 2019). One of the latest ACT assays available on the market as POC analyser, the i‐STAT 1 (Abbott Point‐of‐Care, Wavre, Belgium), uses an amperometric sensor to detect an alternative test endpoint, namely an electrochemical change that occurs during thrombin cleavage in the sample. Studies in humans suggest that the amperometric measurement improved test reliability in human patients undergoing cardiopulmonary bypass compared to mechanical clot detection (Falter et al., 2018; Thompson et al., 2019). Additional benefits of this analyser are its ease of use, low influence of operator technique and its ability to measure ACT on very low sample volumes (40 μL) (Thompson et al., 2019).

So far, published ACT reference intervals (RIs) in healthy adult dogs have been determined using manual ACT tube tests (Byars et al., 1976; Middleton & Watson, 1978; Thompson et al., 2019; Tseng et al., 2001) and less often using adapted ACT tube tests (See et al., 2009) or automated POC analysers (Gerber et al., 1999; Tseng et al., 2001). One study in dogs compared a manual ACT tube test against an automated veterinary POC analyser (SCA 2000 Veterinary Coagulation Analyzer); however, only 15 healthy dogs were included to determine an RI for this analyser (Tseng et al., 2001). Given the heterogeneity of available ACT assays, ACT measurements from different assays cannot be accurately extrapolated (Thompson et al., 2019).

The first objective of the current study was to determine an RI for ACT in healthy adult dogs using a POC analyser and to investigate the need for sex‐, age‐ and bodyweight‐specific RIs. The second objective was to quantify intra‐subject within‐ and between‐day variability. The third objective was to investigate analyser reliability and inter‐analyser agreement (between two identical analysers). The fourth objective was to evaluate if a short delay in sample analysis (of one analytical run) would influence ACT measurements.

## 2 MATERIALS AND METHODS

### 2.1 Animals

The sample population included dogs that underwent a physical and general blood examination for health screening purposes or blood donation (EC2019‐039, DWZ/EV/19/115/75, DC2020H03, EC2020‐009, EC 2020‐061, DWZ/KF/20.1.15/44, EC2021‐031). These dogs were either client‐owned or purpose‐bred research dogs. Blood examination as part of the health screening included serum biochemistry and haematology (including automated platelet count). Written informed consent was obtained in all client‐owned dogs. Purpose‐bred research dogs were Beagles that required blood sampling and physical examination as part of a pre‐anaesthetic health screening (EC2021‐031) or mandatory annual health screening. Adult dogs (>1 year) were prospectively enrolled based on disease‐ and drug‐free history (besides prophylactic drugs against endo‐ and ectoparasites), absence of abnormalities on physical examination and normal serum biochemistry and haematology.

### 2.2 ACT measurements

All ACT measurements were performed on fresh venous non‐coagulated blood collected by venepuncture of the jugular or cephalic vein using a 21‐G needle and syringe. All ACTs were measured using the i‐STAT 1 POC analyser (Abbott Point‐of‐Care, Wavre, Belgium) and compatible kaolin cartridges (i‐STAT ACTk test cartridge; Abbott, Belgium). Cartridges had a measuring range of 50–1000 s. Ten minutes prior to blood sampling, cartridges, which were stored at 4°C, were acclimated at room temperature (18–25°C). ACT values were determined within 15 s of blood collection (unless for samples testing measurement delay) by adding a single drop (±40 μL) of blood from the syringe on the sample port.

### 2.3 Establishment of RI

Based on the American Society for Veterinary Clinical Pathology (ASVCP) guidelines for the determination of RIs, a minimum of 40 dogs is recommended (Friedrichs et al., 2012). Enrolment was performed on a first come, first served basis, but the number of purpose‐bred research dogs was limited to one third of the reference population. Only the first ACT measurement of each dog (T0) was used to determine the RI.

### 2.4 Longitudinal intra‐subject variability

Dogs included in a research project involving healthy client‐owned dogs (EC2020‐009, EC 2020‐061, DWZ/KF/20.1.15/44) were subject to additional serial blood collections: at time zero (T0), 2 h later (T2) and 48 h later (T48); these samples were used for intra‐subject within‐day (T0–T2) and between‐day (T0–T48) variability measurements.

### 2.5 Analyser reliability and inter‐analyser agreement

Both client‐owned dogs and purpose‐bred research dogs were used. Analyser reliability and inter‐analyser agreement were studied by duplicate measurements on two identical analysers under similar conditions. Two cartridges were filled in immediate succession and the analyser receiving the first cartridge was alternated. The total number of duplicate measurements was determined by the availability of suitable study dogs during a 1‐week period when a second analyser was available at our clinic.

### 2.6 Measurement delay

Only purpose‐bred research dogs were used. To study the influence of a short time delay (T0 delay) of one analytical run before performing the analysis and based on the available blood volume, ACT was remeasured directly upon completion of the first run, using the same sample and analyser. The latter would reflect the situation in which ACT is remeasured on the same blood sample due to analyser or cartridge failure.

### 2.7 Data analysis

Statistical analysis was performed using a commercial statistical software package (SPSS 27, IBM, Chicago, Illinois). Normality for baseline ACT (T0), age (months) and weight (kg) was checked using visual inspection of the histogram, quantile‐quantile plots and a Shapiro–Wilk test. For RI estimation, potential outliers were detected by Tukey's method. The ASVCP guidelines recommend the Robust method for RI estimation when less than 120 samples are available, requiring a sample size of ≥40 animals and 90% confidence intervals (CIs). The Robust method was used to establish lower and upper reference limits and corresponding 90% CIs were constructed using a bootstrap method (Reference Value Advisor V2.1, National Veterinary School Toulouse, France). The presence of a linear correlation between baseline ACT (T0) and age or body weight was studied using a Pearson's correlation coefficient *r*. The influence of sex on baseline ACT (T0) was evaluated using an independent‐samples *t*‐test (neutering status was not taken into account considering the size of the subgroups). The presence of a significant difference between T0 and T2, T0 and T48, and T0 and T0 delay was assessed using the Wilcoxon signed rank test. Within‐day (T0–T2) and between‐day (T0–T48) intra‐subject variability was evaluated using the coefficient of variation (CV) computed as intra‐subject standard deviation / mean. The intra‐subject standard deviation was calculated as described earlier (Jones & Payne 1997; Synek, 2008). The result was interpreted as follows—very low variability: <5%; low variability: between 5% and 15%; moderate variability: between 16% and 25%; and high variability: >25% (Bland, 2000). The presence of a difference between identical analysers or inter‐analyser agreement was investigated using the Wilcoxon signed rank test. Analyser reliability was studied by calculating a CV% and intraclass correlation coefficient (ICC) including a 95% CI, obtained from a two‐way mixed model measuring absolute agreement. The ICC is a dimensionless value ranging from 0 to 1 and corresponds to the amount of variability caused by measurement error (1 means no measurement error) (Bartlett & Frost 2008). Interpretation was performed according to Koo and Li (2016)—poor reliability: <0.5; moderate reliability: between 0.5 and 0.75; good reliability: between 0.75 and 0.9; and excellent reliability: >0.90. Results were considered to be statistically significant if the *p*‐value was <0.05.

## 3 RESULTS

### 3.1 Animals

A total of 49 dogs were enrolled. Seven were excluded due to abnormalities on serum biochemistry (*n* = 5), behaviour restricting blood sampling (*n* = 1) or absence of serum biochemistry results (*n* = 1), resulting in a final number of 42 healthy dogs. Eighteen dogs were female neutered, 12 were female intact, eight were male neutered and four were male intact. Dogs had a mean age of 73.1 ± 33.0 months and median body weight of 13.4 kg (range: 2.1–54.0 kg). Seventeen dog breeds were present in the study including Beagle (*n* = 15, one client‐owned Beagle and 14 purpose‐bred research Beagles), Labrador Retriever (*n* = 6), Golden Retriever (*n* = 3), Bernese Mountain Dog (*n* = 2), Chihuahua (*n* = 2), Jack Russel Terrier (*n* = 2), Nova Scotia Duck Tolling Retriever (*n* = 2) and one of each Border Collie, Cavalier King Charles Spaniel, Cross Breed Dog, Kooikerhondje, Lakeland Terrier, Malinois, Maltese, Miniature Schnauzer, Whippet and White Swiss Shepherd Dog.

### 3.2 Establishment of RI

**FIGURE 1:**
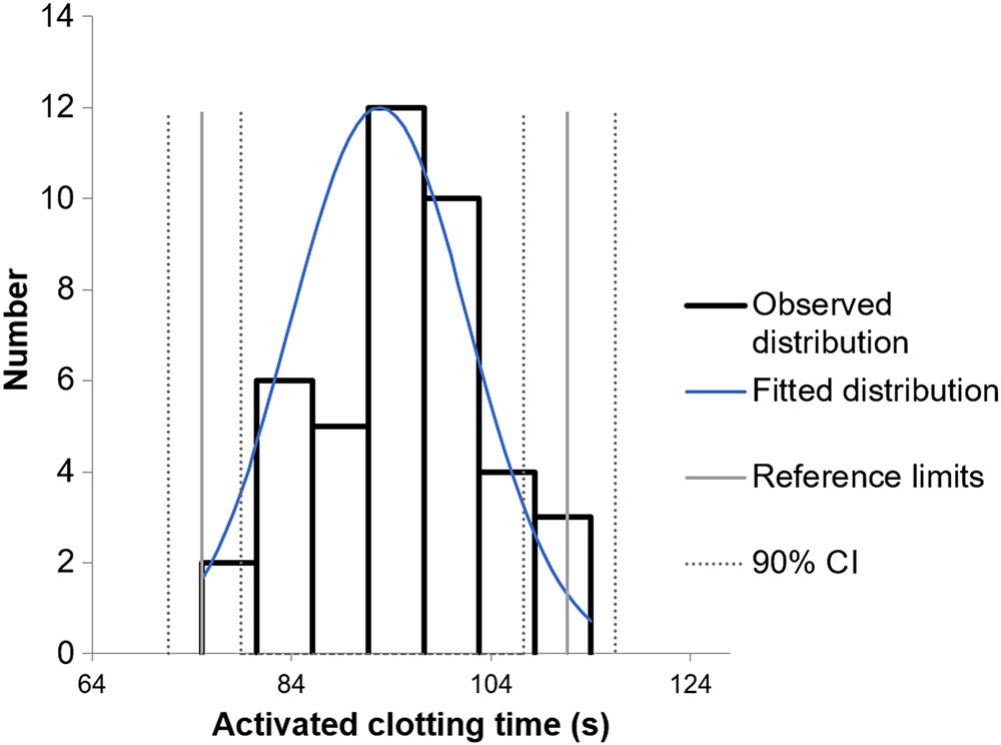
Histogram obtained from 42 healthy dogs displaying a normal distribution for activated clotting time (ACT). Mean ACT was 92.9 ± 9.1 s. Lower and upper ACT reference limits, represented by the two solid vertical grey lines, were 74.4 s (90% CI: 70.1–78.3) and 111.2 s (90% CI: 107.5–115.2), respectively. CI, confidence interval.

Mean ACT in healthy adult dogs was 92.9 ± 9.1 s; lower and upper limit of the RI was 74.4 s (90% CI: 70.1–78.3) and 111.2 s (90% CI: 107.5–115.2), respectively (Figure 1). No significant difference in mean was found between male (96.8 ± 11.8 s) and female dogs (91.3 ± 7.2 s) (*p* = 0.067). Furthermore, no significant linear correlation was observed between ACT and age (*r* = 0.254; *p* = 0.104) or ACT and body weight (*r* = 0.277; *p* = 0.076).

### 3.3 Longitudinal intra‐subject variability

**FIGURE 2:**
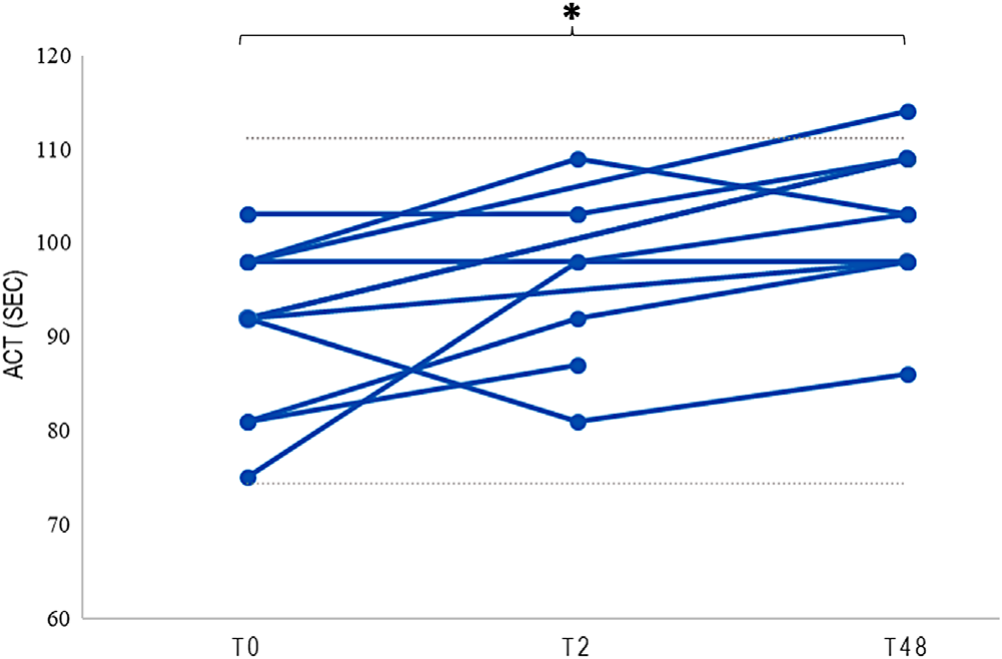
Intra‐subject activated clotting time (ACT) measurements obtained from blood collections at different timepoints: T0 (blood collection and measurement at time zero), T2 (blood collection and measurement 2 h later) and /or T48 (blood collection and measurement 48 h later). A total of eight T2 and 10 T48 ACT samples were available. The grey dotted lines represent the lower limit (74.4 s) and upper limit (111.2 s) of the RI. Whilst no significant within‐day (T0–T2) intra‐subject difference was found (*p* = 0.216; effect size = −0.44), a significant between‐day (T0–T48) intra‐subject difference was present (asterisk) (*p* = 0.020; effect size = −0.74).

Eight T2 and 10 T48 ACT samples (all client‐owned dogs) were available for intra‐subject within‐ and between‐day comparisons, respectively (Figure 2). No significant difference was found for within‐day comparisons (*p* = 0.216; effect size = −0.44), but a statistically significant difference was present for between‐day comparisons (*p* = 0.020; effect size = −0.74) (Table 1). Within‐ and between‐day intra‐subject variability expressed by their respective CV (%) was 8.1 and 10.4, respectively.

**TABLE 1:** Median (range) activated clotting times (ACT) are displayed for the intra‐subject within‐day comparison, the intra‐subject between‐day comparison and the before and after measurement delay comparison.

	Within‐day comparison (*n* = 8)		Between‐day comparison (*n* = 10)		Before and after delay comparison (*n* = 6)	
	T0	T2	T0	T48	T0	T0 delay
ACT measurement (s)	90 (75–103)	98 (81–109)	98 (75–103)	103 (86–114)	90 (87–98)	76 (76–81)
*p*‐value	0.216		0.020		0.027	

*Note*: Measurements were obtained from blood collections/measurements at different timepoints: T0 (blood collection and measurement at time zero), T2 (blood collection and measurement 2 h later), T48 (blood collection and measurement 48 h later) and T0 delay (blood collection at time zero and waiting one analytical run before performing the measurement). The results for the Wilcoxon signed rank test are displayed.

### 3.4 Analyser reliability and inter‐analyser agreement

Inter‐analyser agreement and analyser reliability were studied in eight samples from eight dogs (client‐owned dogs: *n* = 6; purpose‐bred research dogs: *n* = 2). Median ACT for both identical analysers was equal to 103 s (range: Analyser A = 87–109 s; Analyser B = 92–114 s). No significant difference was observed between analysers (*p* = 0.593; effect size = −0.19) (Table 2). In addition, analyser reliability assessed by the CV% and ICC was 3.3% and 0.87 (95% CI: 0.38–0.97), respectively.

**TABLE 2:** Median (range) activated clotting times (ACT) are displayed for the inter‐analyser comparison between two identical point‐of‐care analysers (Analyser A and B).

	Inter‐analyser comparison (*n* = 8)	
	Analyser A	Analyser B
ACT measurement (s)	103 (87–109)	103 (92–114)
*p*‐value	0.593	

*Note*: The result for the Wilcoxon signed rank test is displayed.

### 3.5 Measurement delay

**FIGURE 3:**
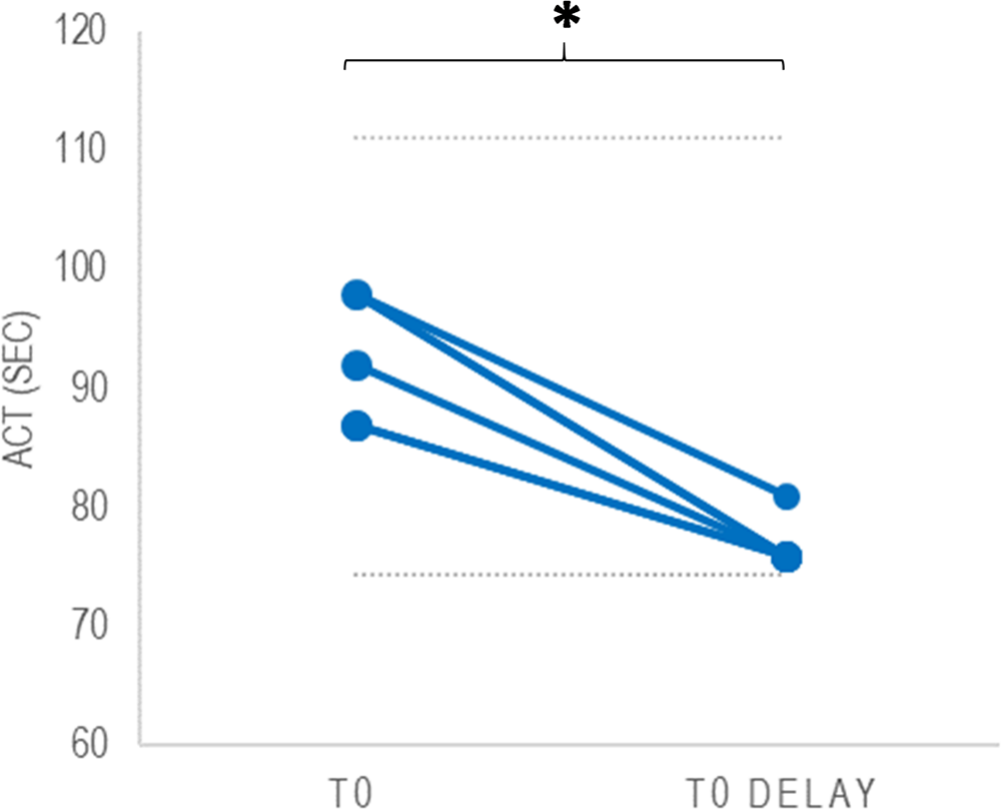
Paired activated clotting time (ACT) measurements performed on the same blood samples both before (T0) and after (T0 delay) waiting one analytical run before performing the measurement. A total of six samples from six dogs were available. The grey dotted lines represent the lower limit (74.4 s) and upper limit (111.2 s) of the RI. Significantly lower ACT values were measured at T0 delay compared to T0 (asterisk) (*p* = 0.027; effect size = −0.90).

Lastly, the effect of a delay in sample processing was studied in six samples from six dogs (all purpose‐bred research dogs) (Figure 3). This revealed significantly lower ACT values after a delay (median T0 delay = 76 s [range: 76–81 s]) compared to direct sample processing (median T0 = 90 s [range: 87–98 s]) (*p* = 0.027; effect size = −0.90) (Table 1).

## 4 DISCUSSION

This study is the first to determine an RI for ACT in healthy adult dogs according to the ASVCP guidelines for RI estimation using the i‐STAT 1 POC analyser. No significant differences between male and female dogs or influence of age and body weight on ACT were observed. Intra‐subject within‐day variability and between‐day variability were considered low. A significant between‐day measurement difference was observed; however, medians of both days were within the established RI. Analyser reliability and inter‐analyser agreement were good. Finally, a delay in sample processing had a significant influence on the ACT measurement.

When comparing our results to most other published RIs for ACT in dogs (Table 3), a higher mean and slightly more narrow range (minimum–maximum) were found. There are a few hypotheses to explain this. Firstly, there are important differences in test methodology and test endpoint between the current POC analyser and all other previously studied assays. For example, the ACT tubes used by See et al. (2009) contained a magnetic indicator that was checked at 5–10 s intervals for the presence of visual, audible and palpable indications for clot formation, which reduced precision and accuracy. Byars et al. (1976) and Middleton and Watson (1978) used visual detection at 5 and10 s intervals, respectively, to confirm clot formation in the test tube. Despite the lack of clearly defined objective endpoints, both tests required multiple sequential manual steps including inverting the test tube and withdrawing the sample from the heating solution, which introduced additional sources of analytical error. Even the automated POC analyser used by Tseng et al. (2001) and Gerber et al. (1999) differed noticeably regarding test endpoint compared to the current analyser. The i‐STAT 1 uses an amperometric sensor to determine the electrochemical changes that occur during thrombin cleavage, which is considered a direct measurement of ACT, while the SCA 2000 Veterinary Coagulation Analyzer (Tseng et al., 2001) and the HemoTec (Gerber et al., 1999) use an optical sensor that recognises either reduced blood flow through a channel or reduced fall velocity of a mechanical plunger when blood coagulation occurs. Both techniques should therefore be considered as an indirect measure of ACT (Falter et al., 2018; Thompson et al., 2019). Secondly, minor differences in pre‐analytical steps (e.g. blood collection, sample handing, operator, etc.) and selection criteria for apparently healthy reference individuals may have played a role. Our study used direct selection methods, including dogs that met predefined biological (age >12 months, any breed and sex) and clinical criteria (disease‐ and drug‐free history, absence of abnormalities on physical examination, serum biochemistry and haematology) and therefore potentially handled more strict inclusion and exclusion criteria than previous studies. Lastly, in both the previous and current study limited sample size may have affected the RIs, as none of the studies described here reached the ideal number of 120 healthy dogs, recommended by the ASVCP guidelines (Friedrichs et al. 2012). A lower number of healthy animals can be used; however, it can result in a higher degree of uncertainty which is reflected by the need for 90% CIs for both lower and upper reference limits (Friedrichs et al. 2012). Previous studies (Table 3) published mean or median ACT and expressed their RI as minimum–maximum or mean ± 2SD which further complicates result comparisons.

**TABLE 3:** Summary of previously reported reference data obtained from healthy dogs using different activated clotting time (ACT) assays.

Assay	Contact activator	Detection method	Mean	Median	SD	Range	*n*	Study
ACT tube	Diatomaceous earth	Visual	77.5	75	14.7	<60–125^a^	72	Byars et al., 1976
ACT tube	Silica	Visual	79.0	n.a.	7.1	64–95	42	Middleton & Watson, 1978
ACT tube	Diatomaceous earth	Visual	103	100	20.3	63–144^b^	27	Tseng et al., 2001
MAX‐ACT tube	Diatomaceous earth, kaolin and glass beads	Visual	71	n.a.	n.a.	55–80^a^	47	See et al., 2009
Medtronic HemoTec	Kaolin	Photo‐optical	79.3	78.5	7.4	64–95^b^	43	Gerber et al., 1999
SCA 2000	Kaolin, silica	Photo‐optical	71	67	11.8	47–95^b^	15	Tseng et al., 2001
i‐STAT 1	Kaolin	Amperometric	92.9	92.8	9.1	74–114^c^	42	Current study

*Note*: Test characteristics are presented on the left, whilst reference data are summarised on the right. All ACT values are expressed in seconds.

Abbreviations: *n*, number of animals in the study; n.a., not available.

^a^ Range = minimum–maximum.

^b^ Range = mean ± 2 standard deviation (SD).

^c^ Range = Robust method.

The influence of subject factors such as sex, age and body weight was studied to investigate the need for specific RIs in these subgroups. None of the three variables seemed to significantly influence ACT, which is in line with earlier studies (Byars et al. 1976; See et al. 2009). Longitudinal biological intra‐subject variability has been quantified for other coagulation parameters in dogs yet remains unstudied for ACT (Wiinberg et al. 2007). Understanding intra‐subject biological variation could be important when performing serial measurements, as it helps clinicians to set a critical difference or threshold above which a change should be considered significant and not caused by biological variation only (Wiinberg et al. 2007). An important side note for further interpretation is that both intra‐subject within‐day variability and between‐day variability reported here are measures of total variability, which is the sum of analytical variability from the measuring analyser and biological variability from the subject. For most automated POC analysers, it mainly includes biological variability (Banfi & Del Fabbro 2009). The CV% for both intra‐subject within‐ and between‐day variability was interpreted as low variability (Bland, 2000). The clinical relevance of the observed between‐day difference was limited, as the medians of both days fell within the RI for healthy dogs.

Analyser reliability and inter‐analyser agreement between identical POC analysers were interpreted as good and comparable to results reported in humans (Koo & Li, 2016; Schussler et al. 2004). However, samples should be analysed directly on fresh blood samples and any delay in sample processing should be avoided. The most likely explanation for lower ACT values after measurement delay is activation of the coagulation cascade before adding the blood to the cartridge. As such, it is advisable to have the analyser available near the patient.

Our study had several limitations. First, the study was limited by the sample size. Despite not reaching the ideally suggested number of 120 samples, our study is the first to determine an RI for ACT according to the recommendations of the ASVCP (Friedrichs et al. 2012). The same sample size limitation should be taken into account when interpreting the results for longitudinal intra‐subject variability, analyser reliability and the effect of a measurement delay. Usually, 10–15 animals or more are advised for this purpose, which was not achieved in all cases in our study (Freeman et al., 2017). The low number of duplications for most comparisons is due to the fact that ACT needs to be measured immediately. Therefore, it was impossible to perform multiple repeated measurements on one blood sample without using multiple analysers or multiple blood draws from the same dog. Still, our study provides valuable indications which could serve as starting point for future research. Second, dogs were assumed to be free of coagulation abnormalities based on the absence of signs of bleeding in the dog's history and during blood sampling, and a normal haematologic panel including automated platelet count. However, coagulation was not assessed using a standard coagulation panel. Third, there was an uneven distribution between the sexes. Therefore, we cannot completely exclude a significant influence of sex, and ideally a larger population with equal distribution between male and female dogs may answer this question. However, previous studies also did not find an influence of sex on ACT (Byars et al. 1976; See et al. 2009). Fourth, there was an uneven distribution in breeds, as about one third of the dogs were purpose‐bred Beagle dogs. Ideally, a more homogeneous distribution of dog breeds is used for RI determination or alternatively separate RIs can be calculated for separate breeds. And finally, when studying the effect of a delay in sample processing, the duration of the delay (one analytical run) was variable and determined by the time required for the first analysis.

In conclusion, our study is the first to provide an RI for ACT in adult dogs according to the ASVCP guidelines. Given the wide array of commercial ACT assays, measurements obtained from different analysers using different techniques should not be interpreted interchangeably. Our initial findings suggest low intra‐subject within‐ and between‐day variability, good analyser reliability and inter‐analyser agreement. To minimise the effect of pre‐analytical variation, it is advised to measure ACT directly upon blood collection. Further research on the use of the current analyser in dogs receiving heparin is required.

## AUTHOR CONTRIBUTIONS

Everyone listed as an author fulfils all three of the ICMJE guidelines for authorship. All authors contributed to the research design. Arnaut Hellemans and Nausikaa Devriendt collected and analysed all data. Arnaut Hellemans wrote the manuscript with input from all authors. All authors have given their final approval to this manuscript. All authors agree to be responsible for all aspects of the work.

## CONFLICT OF INTEREST STATEMENT

The authors declare no conflicts of interest.

## ETHICS STATEMENT

This study has been approved by the Ethical Committee of the Faculty of Veterinary Medicine (Ghent University) and Deontological Committee (EC2019‐039, DWZ/EV/19/115/75, DC2020H03, EC2020‐009, EC 2020‐061, DWZ/KF/20.1.15/44, EC2021‐031).

### PEER REVIEW

The peer review history for this article is available at https://publons.com/publon/10.1002/vms3.1148.

## Data Availability

The data that support the findings of this study are available from the corresponding author upon reasonable request.
